# The circular RNA FAM169A functions as a competitive endogenous RNA and regulates intervertebral disc degeneration by targeting miR-583 and BTRC

**DOI:** 10.1038/s41419-020-2543-8

**Published:** 2020-05-04

**Authors:** Wei Guo, Kun Mu, Bin Zhang, Chao Sun, Ling Zhao, Zhan-Yin Dong, Qing Cui

**Affiliations:** 1 Department of Orthopaedics, Hebei Province Cangzhou Hospital of Integrated Traditional and Western Medicine (Cangzhou No. 2 Hospital), 31 Huanghe Road, Cangzhou, 061001 Hebei PR China; 2 Department of Breast Surgery, Hebei Province Cangzhou Hospital of Integrated Traditional and Western Medicine (Cangzhou No. 2 Hospital), 31 Huanghe Road, Cangzhou, 061001 Hebei PR China; 3 Department of Orthopaedics, Tianjin Medical University General Hospital, 154 Anshan Road, Heping District, Tianjin, 300052 PR China

**Keywords:** Cell biology, Diseases

## Abstract

Intervertebral disc degeneration (IDD) is an important factor leading to low back pain, although the underlying mechanisms remain poorly understood. In this study we examined the role of circular RNA FAM169A (circ-FAM169A) in degenerative nucleus pulposus (NP) tissues, and validated its function in cultured human NP cells. Overexpression of circ-FAM169A in NP cells markedly enhanced extracellular matrix (ECM) catabolism and suppressed ECM anabolism in NP cells. Furthermore, circ-FAM169A sequestered miR-583, which could potentially upregulate BTRC, an inducer of the NF-κB signaling pathway. In conclusion, the present study revealed that circ-FAM169A promotes IDD development via miR-583/BTRC signaling. These findings provide a potential therapeutic option for the treatment of IDD.

## Introduction

Intervertebral disc degeneration (IDD) is a common degenerative disease. IDD features include accelerated extracellular matrix (ECM) degradation and abnormal ECM biosynthesis, decreased hydration, reduced height of intervertebral discs, and suppressed ability to absorb external mechanical compression^1,2^. IDD is the predominant cause of chronic low back pain and spine-related ailments, imposing significant economic and social burdens worldwide^3^. According to previous reports, 84% of the population experience low back pain in their lifetime, with 10% being chronically disabled^4^. Nevertheless, application of current strategies for IDD treatment is hampered by an incomplete understanding of its pathogenesis. To date, IDD treatment is limited to symptomatic interventions, which do not adequately improve outcomes since no disease-modifying drugs are available^5^. Consequently, the clinical management of diseases related to IDD remains severely limited. Therefore, unveiling the pathophysiological events and molecular mechanisms underlying IDD is urgently needed for the development of new treatments.

Intervertebral discs are composed of a central nucleus pulposus (NP), a peripheral annulus fibrosus (AF), and cartilaginous end plates, which connect overlying capillary beds cranially and caudally. NP cells are the predominant cell type in the NP tissue, which forms the main component of the ECM by synthesizing type II collagen (collagen II) and aggrecan, the main functional components of intervertebral discs, to keep the disc height and absorb various mechanical loads. Multiple adverse factors enhance the levels of inflammatory cytokines in the NP, including interleukin-1β (IL-1β) and tumor necrosis factor-α (TNF-α)^6,7^. IL-1β contributes to IDD development by accelerating the degradation of ECM components, for example by increasing the production of catabolic factors such as matrix metalloproteinases (MMPs)^6,7^. Meanwhile, TNF-α influences catabolic pathways in a manner similar to IL-1β. These inflammatory cytokines have been shown to induce an imbalance between anabolic and catabolic activities in NP cells, and to inhibit the expression of anabolic factors (e.g., collagen II and aggrecan), which initiate or accelerate the development of IDD^6,8–10^. Thus, it is necessary to develop an effective way to attenuate inflammatory reactions and reverse the imbalance between anabolism and catabolism within the NP microenvironment.

Multiple molecular inducers of IDD have been recently reported, with non-coding RNAs emerging as key factors affecting IDD pathogenesis^11,12^. Several types of non-coding RNAs have been described, including linear RNAs (microRNAs and long non-coding RNAs) and circular RNAs (circRNAs), which consist of a closed continuous loop^13^. CircRNAs act as post-transcriptional regulators and can interact with microRNAs (miRNAs) via miRNA sponges and competitive endogenous RNAs in the cytoplasm^14–16^. MiRNA sponges are circRNAs with miRNA-binding capacity that absorb miRNAs and inhibit their repressive effects on their targets. Multiple circRNAs have been attributed diverse functions in IDD. While circVMA21, circ-4099, circSEMA4B, and circERCC2 show protective roles in IDD^17–20^, circRNA_104670 was shown to worsen this ailment^21^.

In this study, circRNA (GSE67566), miRNA (GSE63492) and mRNA (GSE56081) expression data sets were downloaded from the Gene Expression Omnibus database (http://www.ncbi.nlm.nih.gov/geo)^22^ for a comprehensive bioinformatic analysis. As a result, we identified several IDD-specific circRNAs and found that circ-FAM169A was significantly upregulated in degenerative NP tissues compared with normal NP tissues. Subsequently, we systematically validated the role of circ-FAM169A in cultured human NP cells.

## Results

### Identification of differentially expressed circRNAs

**Fig. 1:**
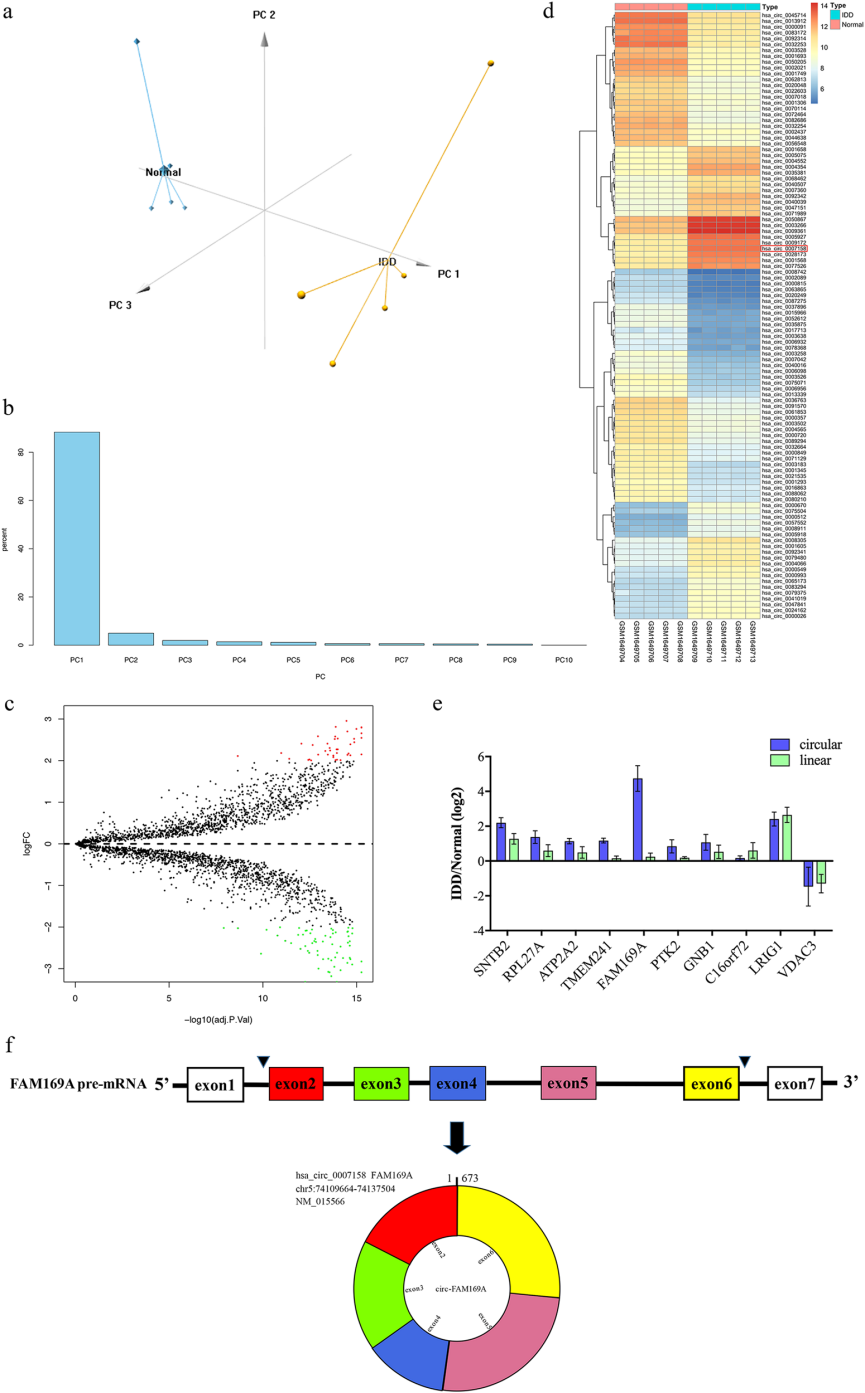
Identification of differentially expressed circRNAs. **a** Amount of variance captured by PCA. **b** PCA score plot. **c** A Volcano plots were used for visualizing differential expression between two different conditions. The horizontal lines correspond to 2.0-fold (log2 scaled) up- and down-regulations, respectively; the vertical line represents an adjusted *P* value of 0.05 (−log10 scaled). The red and green points represent the upregulated- and downregulated circRNAs, with statistical significance. **d** Hierarchical cluster analysis of the significantly upregulated and downregulated-circRNAs. Each column represents a sample, and each row a circRNA. Red, upregulation; green, downregulation. **e** qRT-PCR analysis showing the expression levels of indicated circRNAs and linear isoforms between IDD and normal NP samples. **f** CircFAM169A configuration on chromosome 5 (upper part) showing exons 1–7 that form the precursor (FAM169A pre-mRNA). Its mature, circular form is shown in the lower part of the panel, and includes exons 2–6.

After data normalization, principal component analysis (PCA) of circRNA microarray data was performed (Fig. 1a). Figure 1b shows that the first PC captured more than 88.33% of the total variance in the data while the second and third PCs captured 4.98% and 1.95% of the total variance in the data, respectively. Therefore, only PC1 was further taken into account. As shown in Fig. 1c, the volcano plot identified significantly differentially regulated circRNAs between the two groups. There were 104 differentially expressed circRNAs: 41 circRNAs were upregulated while 63 were downregulated (Supplementary Table S1). Hierarchical clustering showed that circRNA expression patterns were distinguishable between IDD and normal control samples (Fig. 1d). Table 1 shows the top10 upregulated circRNAs. Further analysis confirmed that among the ten upregulated circRNAs, circ-FAM169A was most enriched in IDD NP samples (Fig. 1e). The basic information of circFAM169A was shown in Fig. 1f.

**Table 1:** Top10 upregulated circRNAs in IDD samples.

circRNAs	circBase ID	logFC	adj.pValue
circ-SNTB2	hsa_circ_0040039	2.95291848	3.49E–15
circ-RPL27A	hsa_circ_0092342	2.807559	1.30E–14
circ-ATP2A2	hsa_circ_0028173	2.79475428	5.49E–16
circ-TMEM241	hsa_circ_0047151	2.75996626	1.03E–15
circ-FAM169A	hsa_circ_0007158	2.70014862	3.14E–15
circ-PTK2	hsa_circ_0008305	2.65459631	5.49E–16
circ-GNB1	hsa_circ_0009361	2.58136682	1.16E–13
circ-C16orf72	hsa_circ_0000670	2.5804543	1.39E–14
circ-LRIG1	hsa_circ_0003266	2.54457692	5.49E–16
circ-VDAC3	hsa_circ_0005927	2.52944392	1.02E–14

### Circ-FAM169A is upregulated in degenerative NP tissues and regulates the synthesis of ECM components

**Fig. 2:**
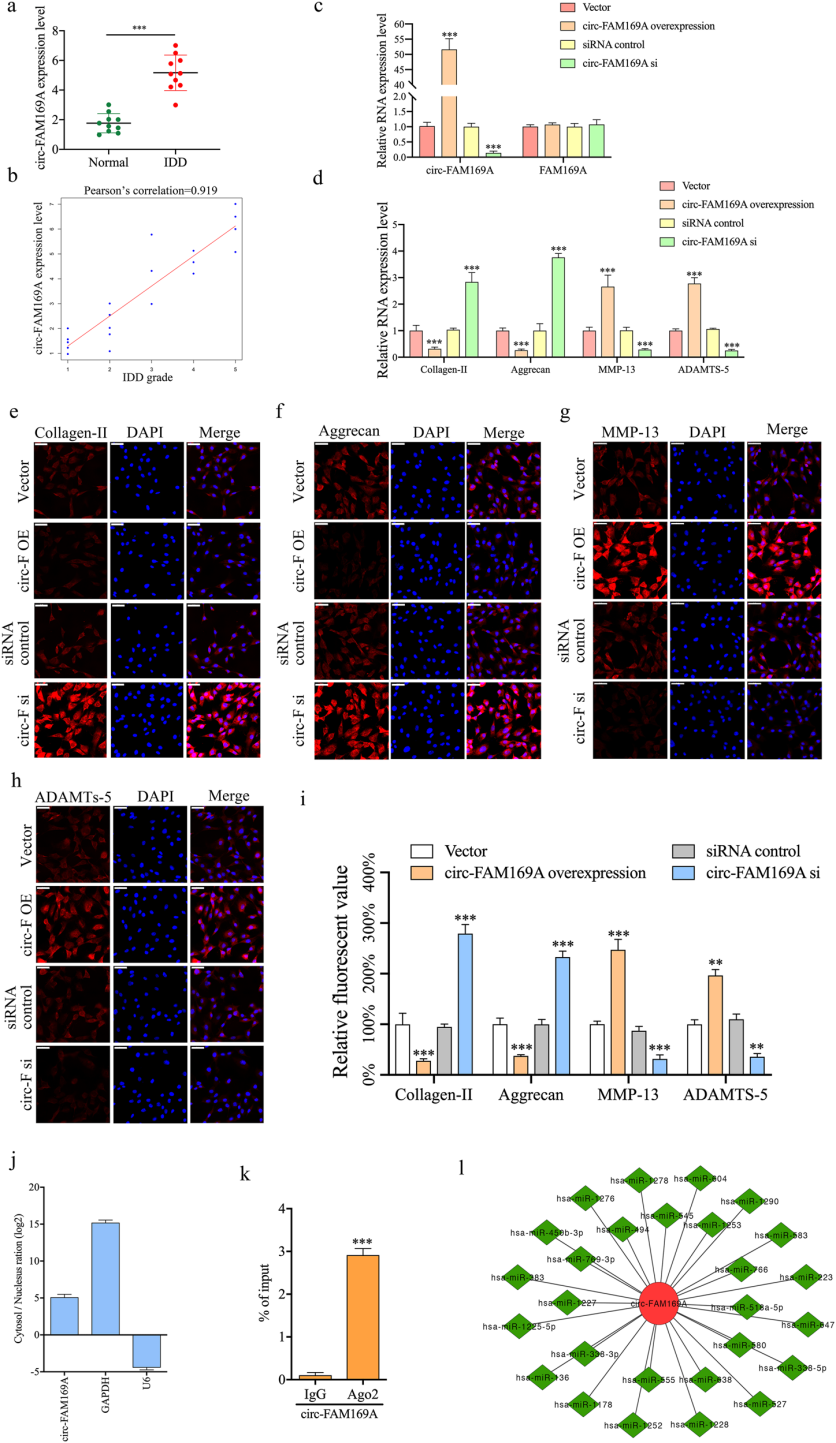
Circ-FAM169A is upregulated in degenerative NP tissues and regulates the synthesis of ECM components. **a** The expression levels of circ-FAM169A in NP tissues were measured in 10 patients and 10 controls using qRT-PCR (***P < 0.001). **b** Circ-FAM169A expression had a significant positive correlation with IDD grade. **c** NP cells were transfected with circ-FAM169A siRNA or circ-FAM169A siRNA control. The levels of circ-FAM169A and its linear counterpart in NP cells were analyzed by qRT-PCR (***P < 0.001). **d** The expression levels of collagen-II, aggrecan, MMP13 and ADAMTS-5 were detected by qRT-PCR (***P < 0.001). **e–h** collagen-II, aggrecan, MMP-13, ADAMTS-5 expression levels were analyzed in circ-FAM169A siRNA transfected cultured human NP cells by immunofluorescence. The corresponding bar graphs show quantitative analysis of the relative fluorescent value of each group. Scale bar = 100 μm. **i** The corresponding bar graphs show quantitative analysis of the relative fluorescent value of each group (**P < 0.01, ***P < 0.001). **j** qRT-PCR analysis of cytoplasmic-to-nuclear expression ratios of circ-FAM169A, GAPDH, and U6 in NP cells. **k** RIP analysis of circ-FAM169A in NP cells using antibodies against AGO2. The RIP enrichment of the AGO2-associated circ-FAM169A was measured by qRT-PCR (***P < 0.001). **l** Circ-FAM169A and its 27 target miRNAs.

qRT-PCR demonstrated that circ-FAM169A was significantly upregulated in degenerative NP tissues compared with controls (Fig. 2a). These findings were confirmed by fluorescence in situ hybridization (FISH), as shown in Supplementary Fig. S1, with the IDD group showing significantly increase in fluorescence levels. Pearson correlation analysis revealed that circ-FAM169A was positively correlated with IDD grade (*r* = 0.919, Fig. 2b). Representative MRI data are shown in Supplementary Fig. S2. Next, the functions of circ-FAM169A in NP cells were assessed. Notably, overexpressing or knockdown of circ-FAM169A in NP cells only influenced the expression of circRNA and not its linear counterparts, as demonstrated by qRT-PCR (Fig. 2c). Therefore, the effects of circ-FAM169A in NP cells were investigated. Circ-FAM169A overexpressing NP cells demonstrated increased expression of MMP-13 and ADAMTS-5, and decreased expression levels of collagen II and aggrecan. On the other hand, circ-FAM169A knockdown contributed to elevated contents of collagen-II and aggrecan, and decreased amounts of MMP-13 and ADAMTS-5 in NP cells (Fig. 2d). This effect was further confirmed by immunofluorescence (Fig. 2e-i). It is known that circRNAs can act as miRNA sponges to regulate gene expression in different cell types^23^. To determine whether circ-FAM169A regulates IDD through the same mechanism, we first examined the relative expression levels of circ-FAM169A in the cytoplasmic and nuclear compartments of NP cells. qRT-PCR results demonstrated that circ-FAM169A was enriched in the cytoplasm (Fig. 2j). RIP assays further showed that circ-FAM169A was enriched in AGO2 immunoprecipitates (Fig. 2k), suggesting that circ-FAM169A possessed miRNA-related functions. To determine which miRNAs potentially interact with circ-FAM169A, we analyzed putative miRNA-targeting sites on the circ-FAM169A sequence using CircInteractome, a web tool for exploring circular RNAs and their interacting miRNAs. We found 30 sites that were targeted by 27 different miRNAs (Fig. 2l)

### Circ-FAM169A acts as a miR-583 sponge

**Fig. 3:**
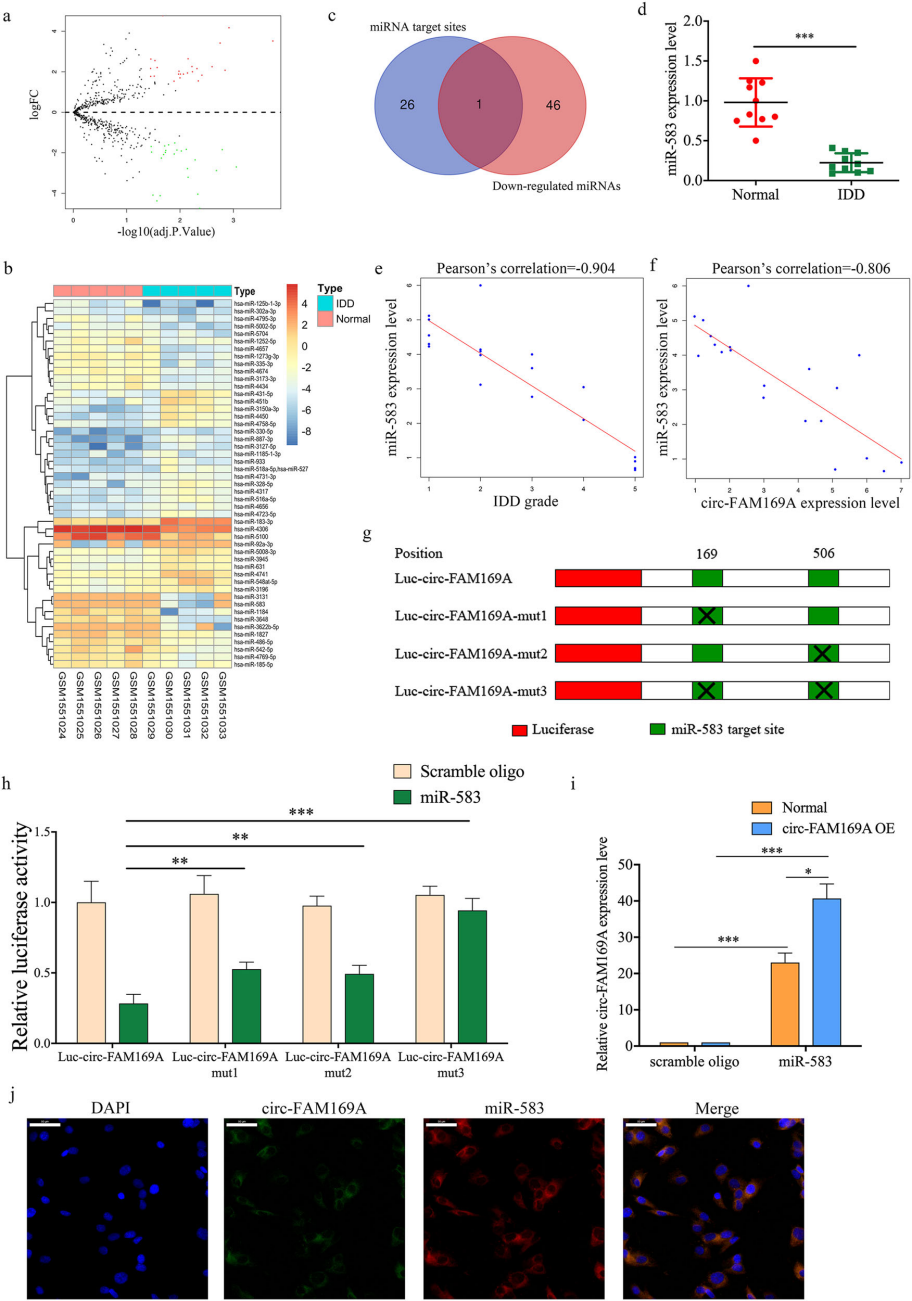
Circ-FAM169A acts as a miR-583 sponge. **a** A Volcano plots were used for visualizing differential expression between two different conditions. The horizontal lines correspond to 1.5-fold (log2 scaled) up- and down-regulations, respectively; the vertical line represents an adjusted *P* value of 0.05 (−log10 scaled). The red and green points represent upregulated- and downregulated-expressed miRNAs, with statistical significance. **b** Hierarchical cluster analysis of the significantly upregulated and downregulated miRNAs. Each column represents a sample, and each row a miRNA. Red, upregulation; green, downregulation. **c** Venn diagram demonstrating the intersection of downregulated miRNAs and predicted target miRNAs. d The expression levels of miR-583 in NP tissues were measured in 10 patients and 10 controls by qRT-PCR (***P < 0.001). **e** MiR-583 expression showed a significant negative correlation with IDD grade. **f** MiR-583 expression had a significant negative correlation with circ-FAM169A expression. **g** Schematic illustration showing the 3′ UTR of luciferase reporters containing the complete circ-FAM169A sequence (Luc-circ-FAM169A) and circ-FAM169A sequence with deletions of miR-583 (Luc-circ-FAM169A-mut1, Luc-circ-FAM169A-mut2 and Luc-circ-FAM169A-mut3) binding sites, respectively. **h** Reporter assays showing the luciferase activities of Luc-circ-FAM169A and Luc-circ-FAM169A-mut1-3 in NP cells co-transfected with miR-583 mimics, or a scrambled oligonucleotide (control) (**P < 0.01, ***P < 0.001). **i** qRT-PCR analysis of circ-FAM169A pulled-down by biotinylated miR-583 mimics in NP cells (*P < 0.05, ***P < 0.001). **j** FISH detection of the subcellular localizations of circ-FAM169A and miR-583. Both molecules were co-localized and both cytoplasmic.

After data normalization, 49 differentially expressed miRNAs were detected from NP cell expression profile: 25 miRNAs were upregulated and 24 were downregulated (Supplementary Table S2). The volcano plot identified significantly differentially changed miRNAs between the two groups (Fig. 3a). Hierarchical clustering showed that miRNAs expression patterns were distinguishable between IDD and normal samples (Fig. 3b). The Venn diagram revealed the intersection of downregulated miRNAs and predicted miRNAs (Fig. 3c). Notably, miR-583 was the only one downregulated miRNA potentially interacting with circ-FAM169A. MiR-583 expression levels were further validated by qRT-PCR in ten pairs of samples. Figure 3d shows that miR-583 was significantly downregulated in IDD NP tissues. Moreover, miR-583 was negatively correlated with IDD grade (*r* = −0.904, Fig. 3e). Interestingly, circ-FAM169A and miR-583 demonstrated a significant negative correlation (*r* = −0.806, Fig. 3f). Therefore, we hypothesized that circ-FAM169A may function as a miR-583 sponge in NP cells. Luciferase reporter assays showed that miR-583 expression significantly reduced the luciferase activity of Luc-circ-FAM169A (a reporter containing the complete circ-FAM169A sequence appended to the 3′-untranslated region of luciferase), whereas the luciferase activity of Luc-circ-FAM169A-mut1-3 containing mutated miR-583 binding sites was not noticeably affected (Fig. 3g–h). Pull-down assay revealed that circ-FAM169A was enriched in the precipitates of miR-583 captured fraction compared with precipitates of scrambled oligonucleotide (Fig. 3i). Moreover, FISH assays demonstrated that circ-FAM169A and miR-583 were co-localized and both cytoplasmic (Fig. 3j). Taken together, these findings demonstrated that circ-FAM169A could directly bind to miR-583 in NP cells.

### Function of miR-583 in NP cells

**Fig. 4:**
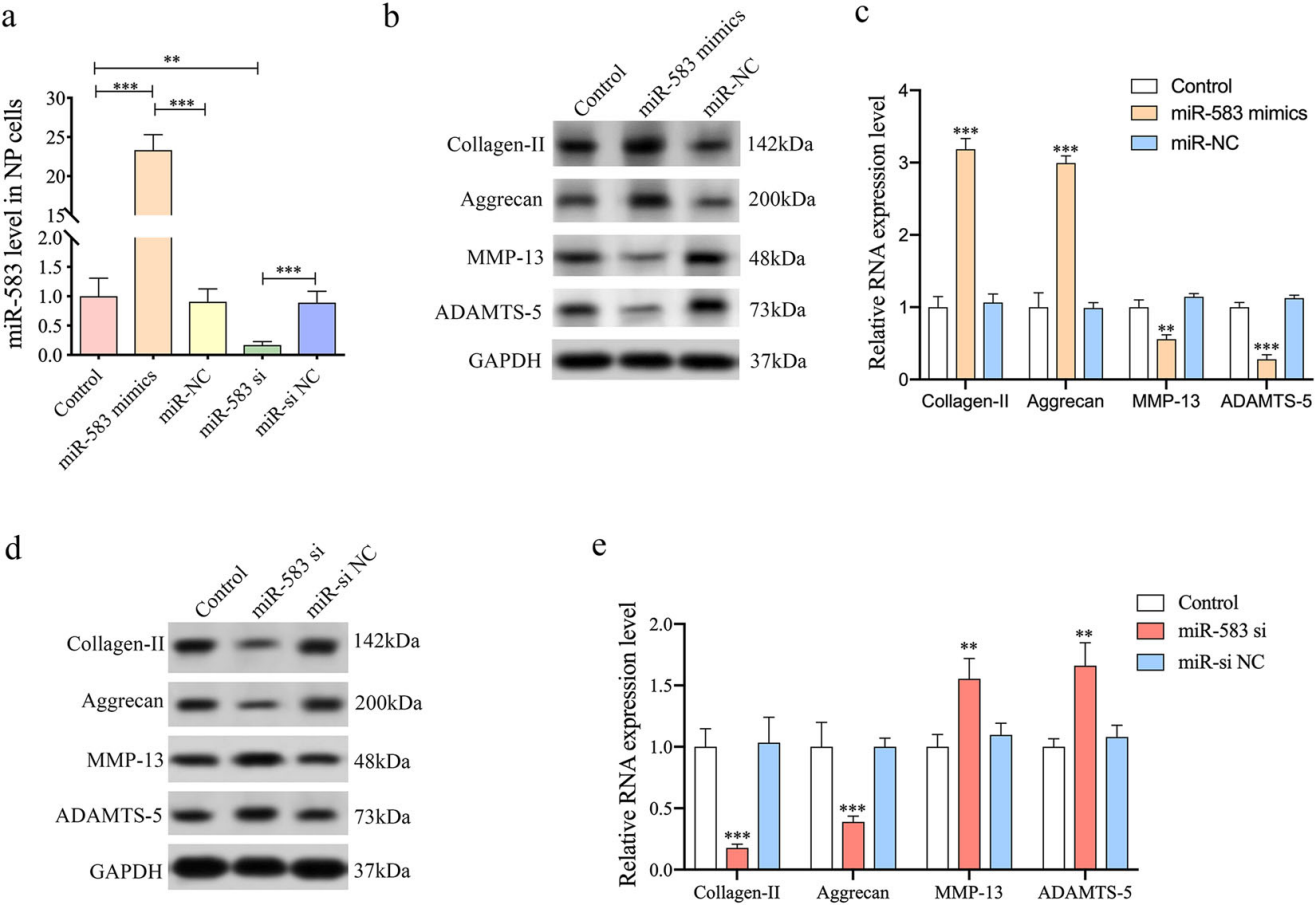
Function of miR-583 in NP cells. **a** NP cells were transfected with miR-583 mimics, miR-NC, miR-583 siRNA, or miR-si NC. Then, miR-583 levels in NP cells were analyzed by qRT-PCR (**P < 0.01, ***P < 0.001). **b**, **c** Western blotting and qRT-PCR were performed to analyze collagen-II, aggrecan, MMP-13, ADAMTS-5 protein and mRNA expression levels, respectively, in NP cells after transfection with miR-583 mimics (**P < 0.01, ***P < 0.001). **d**, **e** Western blotting and qRT-PCR were performed to analyze the collagen-II, aggrecan, MMP-13, ADAMTS-5 protein, and mRNA expression levels, respectively, in NP cells after transfection with miR-583 siRNA. (**P < 0.01, ***P < 0.001).

To further assess the function of miR-583, we overexpressed or knockdown miR-583 in NP cells (Fig. 4a). Cultured human NP cells were transfected with miR-583 mimics or their negative control, respectively. Overexpressing miR-583 resulted in increased contents of collagen-II and aggrecan in NP cells and decreased expression levels of MMP-13 and ADAMTS-5 (Fig. 4b, c). In contrast, the expression levels of collagen-II and aggrecan were reduced in miR-583 knockdown NP cells and MMP-13 and ADAMTS-5 were upregulated (Fig. 4d, e).

### Identification of BTRC as a target gene of miR-583

**Fig. 5:**
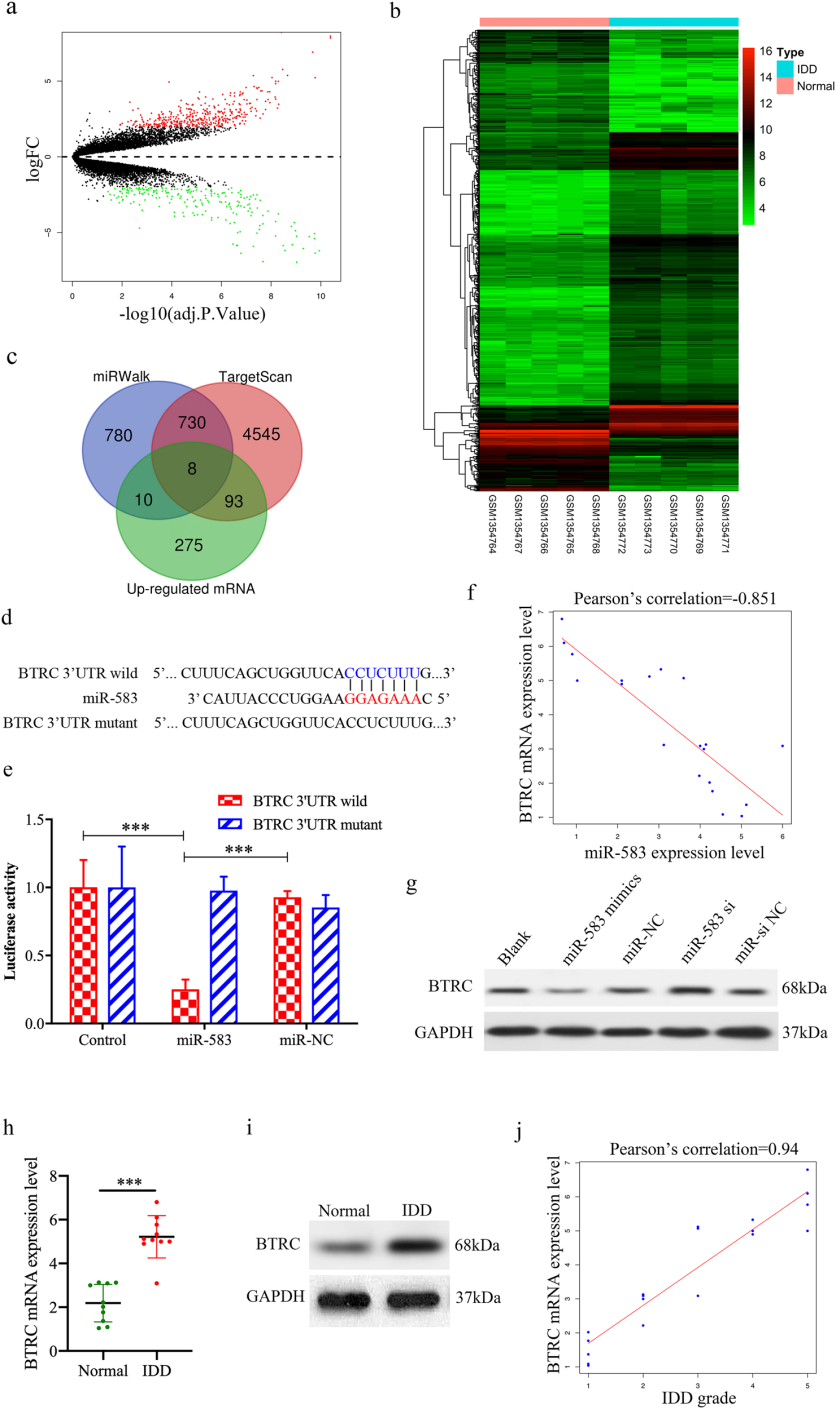
Identification of BTRC as a target gene of miR-583. **a** Volcano plots were used for visualizing differential expression between two different conditions. The horizontal lines correspond to 2.0-fold (log2 scaled) up- and down-regulations, respectively; the vertical line represents an adjusted *P* value of 0.05 (−log10 scaled). The red and green points represent upregulated- and downregulated-expressed mRNAs, with statistical significance. **b** Hierarchical cluster analysis of the significantly upregulated and downregulated mRNAs. Each column represents a sample and each row represents an mRNA. Red, upregulation; green, downregulation. **c** The Venn diagram indicates the intersection of upregulated mRNAs and target mRNA predicted by the miRWalk and TargetScan databases. **d** Sequence alignment of human miR-583 and the 3′-UTR region of BTRC mRNA. Bottom, mutations in the 3′-UTR region of BTRC to create mutant luciferase reporter constructs. **e** Luciferase reporter assay in NP cells after transfection with miR-NC or miR-583 mimics (***P < 0.001). **f** BTRC expression had a significant negative correlation with miR-583 expression. **g** NP cells were transfected with miR-583 mimics, miR-NC, miR-583 siRNA, or miR-si NC. Western blot analysis showed that BTRC expression was suppressed by miR-583 upregulation and elevated by miR-583 knockdown. **h**, **i** The expression of BTRC levels in NP tissues were measured in 10 patients and 10 controls by qRT-PCR and Western blot (***P < 0.001). **j** BTRC expression had a significant positive correlation with IDD grade.

To investigate the functional effects of circ-FAM169A/miR-583, we further assessed the mRNA expression data set, and 599 differentially expressed mRNAs were identified from NP cell expression profile: 386 mRNAs were upregulated and 213 were downregulated (Supplementary Table S3). The volcano plot identified significantly differentially changed mRNAs between the two groups (Fig. 5a). Hierarchical clustering showed that mRNA expression patterns were distinguishable between IDD and normal control samples (Fig. 5b). Next, we predicted target genes of miR-583 using the miRWalk and TargetScan databases. The Venn diagram revealed that eight genes (BTRC, SEC22C, CLPTM1L, TREM1, KCTD12, CD99, ZNF521, and MLEC) were simultaneously predicted by both databases, with upregulated mRNAs (Fig. 5c). As predicted by bioinformatic programs, BTRC, a well-known regulator of NF-κB, is a potential target of miR-583 (Fig. 5d). Indeed, transfection with miR-583 mimics significantly decreased the luciferase activity of wild-type BTRC reporter, whereas introducing mutations in the target site abolished this inhibitory effect (Fig. 5e). Pearson correlation analysis revealed that BRTC and miR-583 had a significant negative correlation (*r* = −0.851, Fig. 5f). In addition, overexpression of miR-583 in NP cells resulted in decreased BTRC expression, and knockdown of endogenous miR-583 exerted the opposite effect in NP cells (Fig. 5g). These results indicated that miR-583 regulated NP cell function by targeting BTRC. Moreover, BTRC expression level was markedly increased in IDD patients’ NP samples (Fig. 5h, i). Interestingly, BTRC expression level was positively correlated with IDD grade (*r* = 0.94, Fig. 5j).

### Circ-FAM169A regulates IDD by modulating the BTRC/NF-κB signaling pathway

**Fig. 6:**
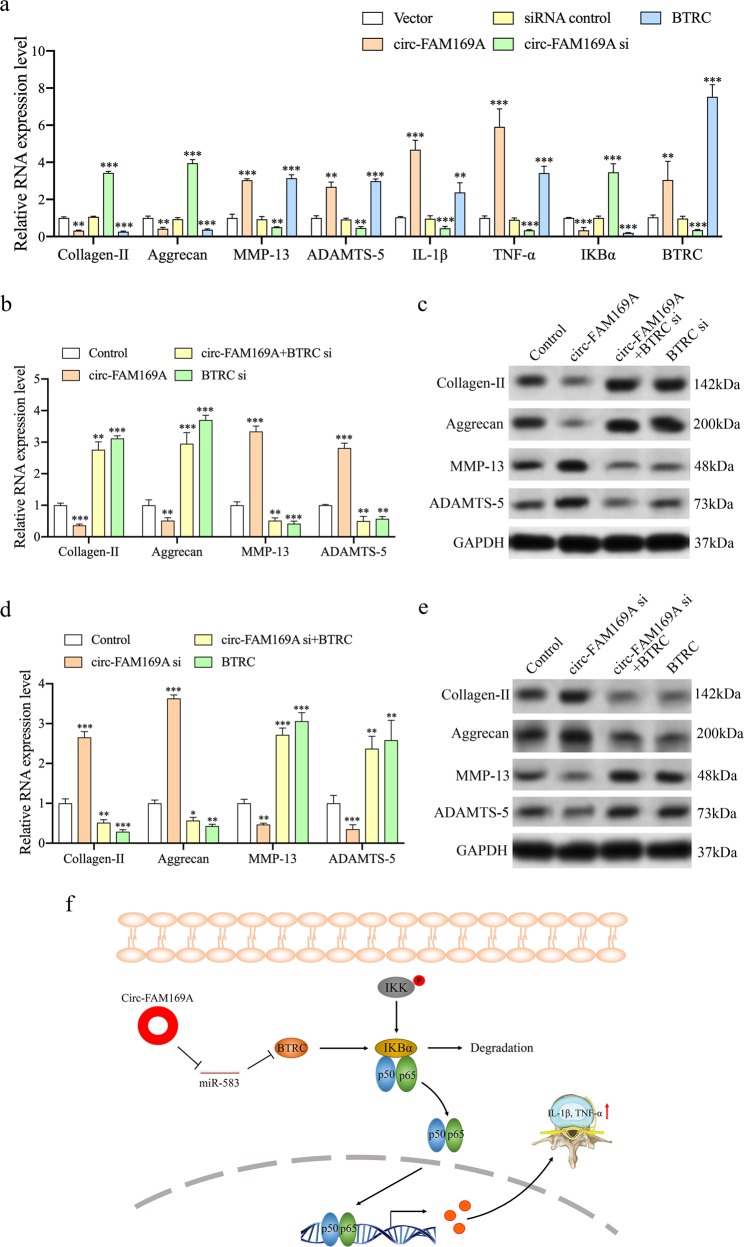
Circ-FAM169A regulates IDD by modulating the BTRC/NF-κB signaling pathway. **a** Cultured NP cells were transfected with circ-FAM169A, circ-FAM169A siRNA, their negative controls, or BTRC for 72 h, and the levels of collagen-II, aggrecan, MMP-13, ADAMTS-5, IL-1β, TNF-α, IKBα and BTRC were measured by qRT-PCR (**P < 0.01, ***P < 0.001). **b**, **c** The rescue experiment was established in cultured NP cells to validate the relationship between circ-FAM169A and BTRC. qRT-PCR and Western blotting demonstrated that reduced collagen-II and aggrecan amounts as well as increased MMP13 and ADAMTS-5 expression levels by circ-FAM169A were blunted by knockdown of BTRC (**P < 0.01, ***P < 0.001). **d**–**e** qRT-PCR and Western blotting showed that upregulation of collagen-II and aggrecan by circ-FAM169A siRNA was abolished by BTRC overexpression. Conversely, downregulation of MMP-13 and ADAMT-5 by circ-FAM169A silencing was abolished by BTRC overexpression (**P < 0.01, ***P < 0.001). **f** Schematic representation of the mechanisms by which circ-FAM169A mediates IDD development. Based on the findings described in the manuscript, circ-FAM169A upregulates BTRC in NP cells, leading to the degradation of IKBα. In turn, this leads to increased levels of multiple proinflammatory cytokines (IL-1β and TNF-α), decreased collagen-II and aggrecan levels, and increased levels of MMP-13 and ADAMTS-5, inducing an imbalance between anabolic and catabolic activities of NP cells. These adverse factors initiate or accelerate IDD.

BTRC is a member of the F-box protein family which mediates the ubiquitination of NFKBIA, NFKBIB, and NFKBIE and their subsequent proteasomal degradation; this degradation frees the associated NF-κB to translocate into the nucleus and to activate transcription. qRT-PCR was used to assess the effect of circ-FAM169A on BTRC expression. Overexpression of circ-FAM169A in cultured human NP cells increased the expression level of BTRC. However, circ-FAM169A upregulation counteracted the inhibitory effect of miR-583 on BTRC expression in NP cells (Supplementary Fig. S3). These findings prompted us to investigate the potential association between circ-FAM169A and BTRC/NF-κB pathway. Cultured human NP cells were transfected with circ-FAM169A, circ-FAM169A siRNA, or their negative controls, respectively. The expression levels of MMP13, ADAMTS-5, IL-1β, TNF-α, and IKBα were significantly increased in NP cells overexpressing circ-FAM169A (Fig. 6a). In contrast, MMP13, ADAMTS-5, IL-1β, TNF-α, and IKBα were downregulated in NP cells transfected with circ-FAM169A siRNA (Fig. 6a). Furthermore, BTRC overexpression affected MMP13, ADAMTS-5, IL-1β, TNF-α, and IKBα genes similar to circ-FAM169A induction (Fig. 6a), indicating that circ-FAM169A regulates IDD progression by targeting the BTRC/NF-κB pathway. Further experiments were performed to validate the relationship between circ-FAM169A and BTRC/NF-κB (Fig. 6b–e). These results indicated that circ-FAM169A mediated IDD progression was primarily through the BTRC/NF-κB pathway (Fig. 6f).

### Therapeutic use of circ-FAM169A silencing in a rat IDD model

**Fig. 7:**
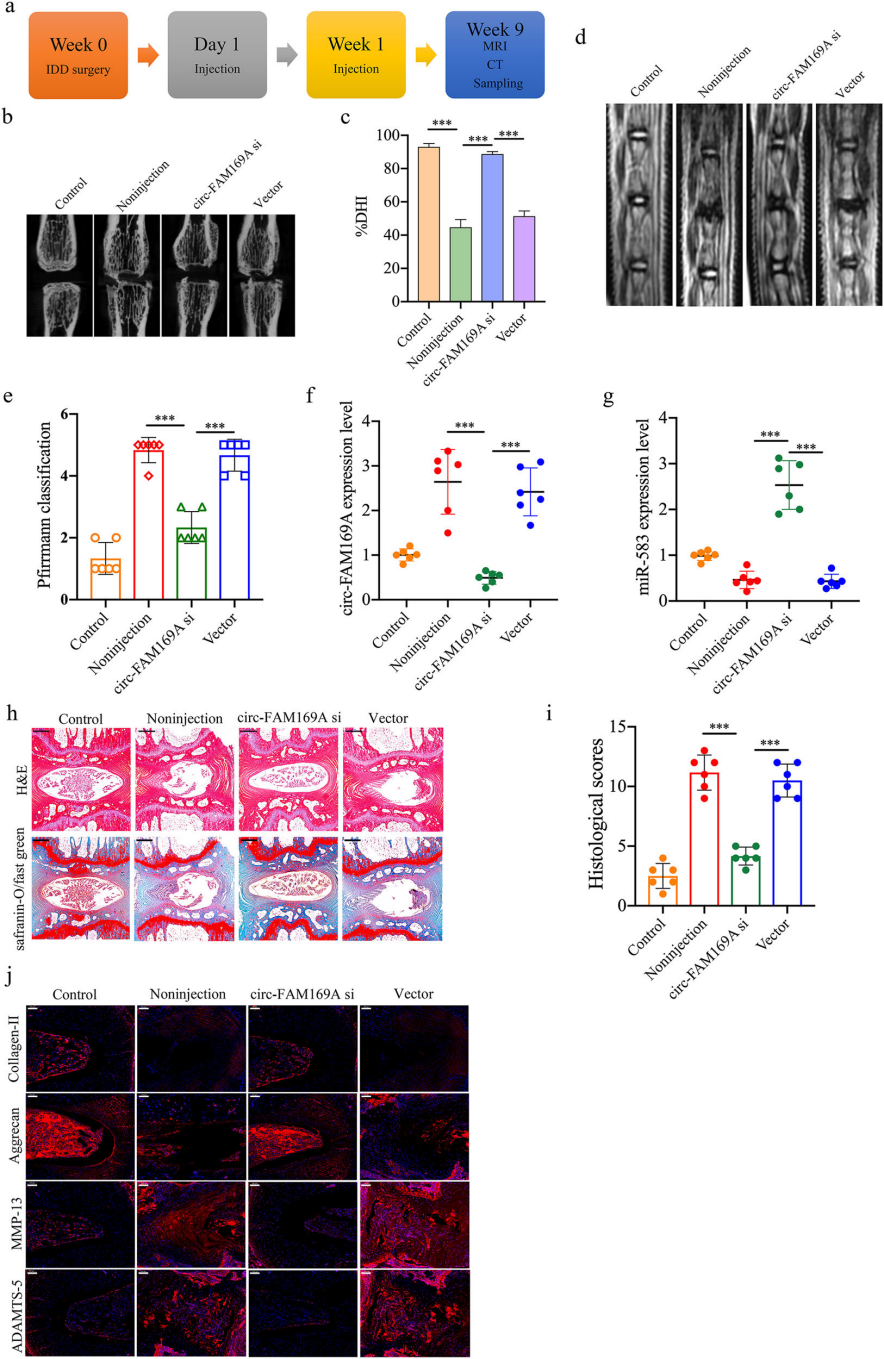
Circ-GRB10 attenuates IDD development in vivo. **a** Overview of the experimental set-up with injections of circ-FAM169A siRNA, or negative control at 1, and 7 days after surgery. **b** CT scans of the indicated groups were obtained 9 weeks after needle puncture. **c** A significant increase in DHI% was noted at 9 weeks post surgery in rats treated with circ-FAM169A siRNA. **d** MRI of the indicated groups were obtained 9 weeks after needle puncture (***P < 0.001). **e** MRI grades in the indicated groups at 9 weeks after needle puncture. The degree of disc degeneration assessed by MRI grade was significantly lower in the circ-FAM169A siRNA group compared with the non-injection group (***P < 0.001). **f** qRT-PCR showed that high circ-FAM169A levels in the punctured intervertebral discs were decreased by the injection of circ-FAM169A siRNA (***P < 0.001). **g** qRT-PCR showed that the decreased levels of miR-583 in the punctured intervertebral discs were rescued by the injection of circ-FAM169A siRNA (***P < 0.001). **h** H&E (top) and safranin-O/fast green (bottom) staining of intervertebral discs in the indicated groups at 9 weeks after needle puncture. Scale bar = 500 μm. **i** A significant decrease in intervertebral disc grade was noted in the circ-FAM169A knockdown group compared with the non-injection group (***P < 0.001). **j** Immunostaining for collagen-II, aggrecan, MMP-13 and ADAMTS-5 in the IDD model treated with circ-FAM169A siRNA at 9 weeks. Scale bar = 100 µm.

We aim to investigate the therapeutic role of circ-FAM169A silencing in IDD and to elucidate the underlying molecular mechanisms. The IDD model was induced in SD rats, followed by local injection of circ-FAM169A inhibitor at 1 and 7 days after surgery with 33-G needles (Fig. 7a). CT scan revealed that the circ-FAM169A inhibitor markedly preserved the intervertebral discs as structurally assessed by radiography (Fig. 7b, c). At 9 weeks after injection, the MRI degeneration score of the intervertebral discs was significantly lower in the circ-FAM169A silencing group compared with the non-injection group (Fig. 7d, e). After injection of adenoviral circ-FAM169A inhibitor, the levels of circ-FAM169A in the degenerative NP tissues were significantly decreased, while the levels of miR-583 were remarkably elevated (Fig. 7f, g). Moreover, histological score was significantly higher in the non-injection group compared with the circ-FAM169A silencing group at 9 weeks. These data indicated significantly decreased intervertebral disc grade after circ-FAM169A silencing (Fig. 7h, i). In this study, the expression levels of collagen-II and aggrecan were significantly increased in the circ-FAM169A inhibitor treatment group, whereas decreased MMP-13 and ADAMTS-5 expression levels were noted (Fig. 7j). Jointly, the above findings suggested a therapeutic role for circ-FAM169A silencing in protecting the discs from degeneration, revealing circ-FAM169A as a candidate therapeutic target in IDD.

## Discussion

Previous studies have indicated that certain circRNAs are dysregulated in the development and progression of IDD and play vital roles by targeting distinct genes that regulate NP cell function^17,18,24^. A key aspect of our study is that we first provided a comprehensive functional and mechanistic characterization of circ-FAM169A in IDD. Dysregulated circ-FAM169A was a ceRNA-regulating BTRC expression by sponging miR-583, which mediates the imbalanced expression of anabolic and catabolic factors of NP cells in IDD. These findings clearly imply that increased circ-FAM169A expression may be a critical factor leading to disc damage in response to pathological conditions. Overall, these findings provide new insights into unrecognized pathophysiological mechanisms underlying disc degeneration.

Accumulating evidence suggests that a variety of cellular events are dysregulated in IDD progression, including NP cell apoptosis, ECM degradation, and proinflammatory cytokine expression^25–28^. Our previous findings have demonstrated that excessive apoptosis of intervertebral disc cells plays a crucial role in IDD pathogenesis^18^. It is currently known that loss of collagen-II is an early sign of IDD^29,30^. Aggrecan, as the main proteoglycan in NP tissues, is critical to normal disc function^31^_ENREF_26^32^. The hallmark of IDD is a progressive loss of the ECM macromolecules, aggrecan and collagen-II. In this study, knockdown of circ-FAM169A markedly enhanced collagen-II and aggrecan levels in NP cells, and decreased the expression levels of MMP-13 and ADAMTS-5 (Fig. 2d–i). These findings suggest that circ-FAM169A might play a role in the development of IDD by influencing ECM composition. We thus aimed to explore the possible mechanisms of circ-FAM169A implicated in IDD pathogenesis. Bioinformatic analysis found circ-FAM169A contained multiple target sites of miR-583, which was validated by luciferase, pull-down, and RNA binding protein immunoprecipitation assays. In addition, BTRC was confirmed as a direct target of miR-583 in present study (Fig. 4d–j). BTRC, a component of the Skp-Cullin-F-box-containing E3 ubiquitin ligase complex, recognizes the NF-κB inhibitor IKBAlA for proteasomal degradation and processing, respectively. BTRC thus plays a critical role in both canonical and non-canonical NF-κB pathway activation. Here we report that BTRC is a regulator of NF-κB activation. Upregulated BTRC in NP cells enhanced binding to its substrates, promoting ubiquitination and degradation of the NF-κB inhibitor IKBAlA, and enabling p65 to translocate to the nucleus and activate transcription. In addition, the present study demonstrated that circ-FAM169A upregulated BTRC by inactivating miR-583 in NP cells (Supplementary Fig. 1). Overexpression or knockdown of circ-FAM169A significantly affected NF-κB pathway-induced inflammatory cytokine (IL-1β and TNF-α) production (Fig. 6a). BTRC silencing remarkably impaired the proinflammatory ability of circ-FAM169A (Fig. 6b–c). IL-1β and TNF-α have been shown to enhance the degradation of ECM components in NP cells^7^ and play central roles in the pathological process of IDD^6,9,33^. Meanwhile, in intervertebral disc degeneration, the NF-κB signaling pathway is gradually induced, which upregulates matrix degrading enzymes, such as MMP and ADAMTS, aggravating ECM degradation^24^. However, these changes in nucleus pulposus cells can be reversed by blocking the activity of NF-κB signaling, suggesting that the latter pathway may play a pivotal role in regulating disc degeneration^34,35^. These findings highlight the tight relationship between inflammatory factors and ECM metabolism in IDD. This study not only function elucidates a mechanism for circ-FAM169A/miR-583/BTRC pathway mediated degeneration of intervertebral disc but also identifies circ-FAM169A as a of the NF-κB proinflammatory signaling pathway.

In summary, this study confirmed that circ-FAM169A promotes NP cell imbalance between ECM anabolism and catabolism via miR-583/BTRC signaling, and possibly through a pathway involving NF-κB. These findings provide a potential therapeutic option for IDD. Nevertheless, the mechanism by which circ-FAM169A levels are increased in IDD remains largely unclear. Further research is warranted for a comprehensive understanding of the role of circ-FAM169A in IDD. More efforts are needed to investigate whether circ-FAM169A could competitively bind to other miRNAs and whether circ-FAM169A could serve as a molecular target for the treatment of IDD.

## Materials and methods

### Ethics statement

This study was approved by the ethics committees of Tianjin Medical University General Hospital and Hebei Province Cangzhou Hospital of Integrated Traditional and Western Medicine. Human NP tissue samples were obtained from patients undergoing surgery at Tianjin Medical University General Hospital and Hebei Province Cangzhou Hospital of Integrated Traditional and Western Medicine. Written informed consent was obtained from all patients for the use of their tissue specimens for research purpose.

### Clinical specimens

Human degenerative NP specimens were obtained from 10 patients with IDD undergoing discectomy. Control samples were obtained from 10 age- and sex-matched patients with fresh traumatic vertebral fracture undergoing decompressive surgery because of neurological deficits. Table 2 presents the characteristics of the patients.

**Table 2:** Clinical features of the study population.

Variable	Normal (*n* = 10)	IDD (*n* = 10)	*P*
Age (years, mean, SD)	36.2 ± 10.4	34.6 ± 10.3	0.733^a^
Body mass index (kg/m^2^)	23.9 ± 2.2	24.0 ± 2.2	0.982^a^
Sex (%)			
Male	7 (70)	5 (50)	0.650^b^
Female	3 (30)	5 (50)	

*SD* standard deviation.

^a^Student’s *t*-test.

^b^Two-sided chi-squared test.

### Microarray data

CircRNA (GSE67566), miRNA (GSE63492) and mRNA (GSE56081) expression data sets were downloaded from the Gene Expression Omnibus database (http://www.ncbi.nlm.nih.gov/geo)^22^. There were five human NP samples derived from patients with IDD and five from cadaveric discs as normal controls. The platforms were GPL19978 Agilent-069978 Arraystar Human CircRNA microarray V1 for circRNAs, GPL19449 Exiqon miRCURY LNA microRNA Array for miRNAs and GPL15314 Arraystar Human mRNA microarray V2.0 (Agilent_033010 Probe Name version). Probe annotation files were also acquired.

### Preprocessing and differential analysis

Raw data were converted into a recognizable format with the package affy of R. Missing values were imputed by a method based on the K nearest neighbors (KNNs). The KNN-based method selects genes with expression profiles similar to the gene of interest to impute missing values^36^. After background correction and data normalization with the median method^37^, differential analysis was performed using the limma package between disc degeneration samples and controls. For statistical analysis and assessing differential expression, limma uses an empirical Bayesian method to moderate the standard errors of the estimated log-fold changes. The basic statistical method used for significance analysis was moderated t-statistic, which is computed for each probe and each contrast. Moderated *t*-statistic yields *P* values in the same way that ordinary *t*-statistic does, except that the degrees of freedom are increased to reflect the greater reliability associated with the smoothed standard errors. Limma provides the topTable and decideTests functions, which summarize the results of the linear model, perform hypothesis tests, and adjust P values for multiple testing. Results included (log) fold changes, standard errors, *t*-statistics, and *P* values^38^.

### Immunofluorescence

Human NP cells grown on cover glass underwent fixation with 4% formalin (20 min) at ambient, permeabilization with 0.1% Triton X-100 and 0.2% Tween-20 in PBS (40 min at ambient), blocking with 2% goat serum (Invitrogen; 1 h), and incubation with anti-collagen-II (1:200; Abcam, Ab34712), anti-Aggrecan (1:500; Abcam, Ab5790), anti-MMP13 (1:50; Abcam, Ab21624), and anti-ADAMT-5 (1:1000; Millipore, MAB4401) primary antibodies, respectively. After washing, the samples further underwent incubation with fluorescein-conjugated secondary antibodies. Images were captured under a fluorescence microscope (Leica).

### Quantitative reverse transcription-PCR (qRT-PCR)

M-MLV reverse transcriptase (Invitrogen) was employed for reverse transcription of total RNA as directed by the manufacturer. The mRNA levels were assessed by SYBR Green-based qPCR. PCR amplification was carried out in 10-μL reactions comprising cDNA (2 μL), 2× master mix (5 μL), forward and reverse primers (10 μM; 0.5 μL), and water (2 μL) at 95 °C (10 min), followed by 40 cycles of 95 °C (10 s) and 60°C (60 s). Meanwhile, miRNA amounts were quantified with the stem-loop miRNA RT-PCR Quantitation kit (GenePharma). For circRNA detection, total RNA samples were treated with or without 3 U/μg of RNase R (Epicenter, USA) at 37 °C for 20 min, and the resulting RNA subsequently underwent purification with RNeasy MinElute Cleanup Kit (Qiagen). Specific divergent primers for the back-splice junction of circ-GRB10 were used to amplify the circRNA. The resulting amplification products were detected by agarose gel electrophoresis and sequencing. All primers used in this study are listed in Table 3. The relative expression of each sample was determined by the 2^−ΔΔCt^ method^39^.

**Table 3:** Primers sequences used in this study.

Name		Sequence (5ʹ-3ʹ)	
circ-FAM169A			
Forward		TCTCTCATGTATACAGAGGATG	
Reverse		GGCTAATAGGAATCGTAATATTG	
miR-583			
Forward		CAAAGAGGAAGGTCCCATTAC	
Reverse		CAGTGCGTGTCGTGGAGT	
BTRC			
Forward		CCAGACTCTGCTTAAACCAAGAA	
Reverse		GGGCACAATCATACTGGAAGTG	
GAPDH			
Forward		GCACCGTCAAGGCTGAGAAC	
Reverse		GGATCTCGCTCCTGGAAGATG	
U6			
Forward		CTCGCTTCGGCAGCACA	
Reverse		AACGCTTCACGAATTTGCGT	

### Isolation and culture of human NP cells

The NP was separated from AF samples under a stereotactic microscope and sliced at 2–3 mm ^3^, as previously described^18^. NP cells were obtained after digestion with 0.25 mg/mL type II collagenase (Invitrogen) for 12 h at 37°C in Dulbecco’s modified Eagle’s medium/nutrient mixture F-12 (DMEM/F12) (Gibco, Gaithersburg, MD), and resuspended at 2 × 10^5^ cells/mL in DMEM/F12 supplemented with 10% fetal bovine serum (Gibco), 100 mg/mL streptomycin, 100 U/mL penicillin, and 1% L-glutamine. Cell culture was performed at 37 °C in a humidified atmosphere containing 5% CO_2_. At confluency, the cells were trypsinized and passaged, with medium change every other day. Cells at the second passage were assessed in various experiments. The levels of MMP-2, collagen II, and aggrecan in the NP cell culture medium were quantified using Quantikine ELISA kits (R&D Systems, Minneapolis, MN) according to the manufacturer’s instructions.

### Small interfering RNA and circ-FAM169A overexpression plasmid construction

According to the circRNA sequences of circ-FAM169A (hsa_circ_0007158) in circBase, circ-FAM169A small interfering RNAs (siRNAs; three pairs of sequences) and negative control (NC) siRNA were designed and synthesized by Guangzhou Geenseed Biotech Co. (Guangzhou, China). FAM169A transcription was induced in vitro via construction of a circ-FAM169A overexpression vector. Then, front and back circular frames were generated and added to pLCDH-ciR for transcript circularization. The front and back circular frames comprise endogenous flanking genomic sequences with EcoR I and BamH I restriction sites, respectively^23^. The 673 bp target sequence comprised the EcoR I site, splice acceptor AG, circ-FAM169A sequence, splice donor GT, and BamH I site. The PCR product was cloned between the two frames. A mock vector solely containing a non-sense sequence between both circular frames without circ-FAM169A cDNA was generated. Vector construction was performed by Guangzhou Geenseed Biotech Co.

### Cell transfection

Third-generation NP cells were used for transfection with respective plasmids or siRNAs using Lipofectamine 3000 (Invitrogen) according to the manufacturer’s recommendations and as described previously^40^. The cells were harvested for 48 h after transfection.

### Dual-luciferase reporter assay

The binding site of circ-FAM169A (wild-type or mutated) was inserted into the KpnI and SacI sites of the pGL3 promoter vector (Realgene, Shanghai, China). First, cells were seeded on 24-well plates, followed by transfection with plasmid (80 ng), Renilla luciferase vector pRL-SV40 (5 ng), miR-583 mimics, or scramble oligo (50 nM) using Lipofectamine 3000. To generate the WT BTRC 3’UTR-Luc reporter plasmid (BTRC 3′UTR), a portion of the BTRC 3′UTR comprising the putative miR-583 binding site was submitted to PCR and cloned into psi-CHECKTM-2 (Promega) downstream of firefly luciferase using XhoI and NotI (Thermo). Constructs harboring mutations at the predicted miR-583 binding site in WT BTRC 3′UTR were generated by site-directed mutagenesis with Quick Change Lightning Site-Directed Mutagenesis Kit (Agilent Technologies, USA). Cell collection was performed after 48 h and the Dual-Luciferase Assay System (Promega, Madison, WI) was used for luciferase activity measurements as directed by the manufacturer.

### Western blotting

Western blotting was performed as previously described^18^. Cell lysis was carried out in a buffer containing 0.25 M Tris-HCl, 20% glycerol, 4% sodium dodecyl sulfate (SDS), and 10% mercaptoethanol (pH 6.8) supplemented with protease and phosphatase inhibitors. Equal amounts of total protein (10 µg) were separated on 10–12% SDS-polyacrylamide gels and electro-transferred onto polyvinylidene fluoride membranes. After blocking with 5% non-fat milk in Tris-buffered saline with 0.1% Tween-20 (TBST) at room temperature for 1 h, the membranes were incubated with primary antibodies (1:3000) in TBST containing 5% non-fat milk overnight at 4 °C. Secondary antibodies (1:6000) were added at room temperature for 1 h, and immunoblots were developed using an enhanced chemiluminescence system.

### Fluorescence in situ hybridization (FISH)

FISH was performed to detect the subcellular localizations of circ-FAM169A and miR-583 according to the method described by Vautrot et al.^41^. A FISH probe labeled with Alexa Fluor^®^ 488 for circ-FAM169A (Thermo Fisher Scientific, Waltham, MA) was designed to detect the splice junction of two exons. The probe sequence was 5′-CACCTCAACA AAGCAGCCATCTCGTTTCCCAA-3′. The probe for miR-583 was labeled with cy3, with the 5′-GTAATGGGACCTTCCTCTTTG-3′ sequence.

### RNA immunoprecipitation (RIP)

RIP was carried out with Magna RIP Kit (Millipore) as directed by the manufacturer. Briefly, 2 × 10^7^ NP cells underwent UV-crosslinking at 600 mJ/cm^2^ and lysis with 100 μl RIP lysis buffer with a proteinase inhibitor cocktail (Roche) and RNase inhibitor (Promega). Lysates were incubated with DNase I (Roche) at 37 °C for 10 min and submitted to centrifugation at 12,000 × *g* for 30 min. The lysates were next mixed with 900 μl RIP immunoprecipitation buffer and treated for 3 h with 5 μg anti-AGO2 antibodies pre-bound on magnetic beads. An aliquot (10 μl) of this RIP mixture was assessed in parallel. Bead washing (six times) was carried out with RIP wash buffer. Then, 20% of the immunoprecipitate was assessed by immunoblot and the remaining 80% underwent proteinase K treatment at 37 °C for 30 min. RNA extraction was carried out with TRIzol reagent (Invitrogen) as directed by the manufacturer.

### The rat model of IDD

In this study, 48 male Sprague–Dawley rats (3 months) were assessed, adopting the IDD model^42^. In all, 36 rats underwent the surgery and the remaining 12 animals not operated constituted the negative control group. The animals were operated in the prone position following anesthesia (90 mg/kg ketamine and 10 mg/kg xylazine administered intraperitoneally). Under fluoroscopy, 1 intervertebral disc (Co8/9) underwent puncture with 20 G needles; Co7/8 and Co9/10 were untouched as controls. Standard postoperative procedures were carried out.

### Intradiscal injection of circ-FAM169A siRNA

One day following the initial intervertebral disc puncture, the rats were randomized into three groups (non-injection, circ-FAM169A siRNA injection, and Vector injection) with 12 rats/group. After anesthesia, a small incision was made to expose the previously punctured intervertebral disc from the left side. A total of 2 μl solution containing the experimental or control virus vector (~10^6^ plaque-forming units) was slowly injected into the punctured discs with a 33 G needle (Hamilton, Switzerland) attached to a microliter syringe (Hamilton). The injection procedure was repeated at 7 days after IDD surgery.

### Radiography and MRI examination

All rats underwent radiography immediately before the IVD puncture and 8 weeks after the second injection. Disc height was measured using the ImageJ software (US National Institutes of Health) and expressed as the disc height index (DHI) as previously described^43^. Changes in the DHI of the punctured intervertebral discs were expressed as %DHI (%DHI = post-punctured DHI/pre-punctured DHI × 100%). MRI was performed in all rats using a 7.0 T animal specific MRI system (Bruker Pharmascan, Ettlingen, Germany). T2-weighted sections in the median sagittal plane were obtained using the following settings: a fast spin echo (SE) sequence with a time to repetition (TR) of 3000 ms and a time to echo (TE) of 70 ms; the slice thickness was 0.5 mm with a 0 mm gap. The Pfirrmann classification was used to assess the degree of IDD^44^. The average score of the punctured intervertebral discs was calculated as the degeneration grade of each rat.

### Histological evaluation and Immunofluorescent staining

After the MRI examination, rats were sacrificed by intraperitoneal administration of overdose pentobarbital sodium. The samples were fixed with 4% paraformaldehyde for 48 h, decalcified in 10% EDTA, embedded in paraffin, and sectioned (5 μm) along the midsagittal plane. Sections were used for hematoxylin–eosin (H&E) and safranin-O/fast green staining. For Immunofluorescent analysis, antigen retrieval was performed using Antigen-decloaker solution (Biocare Medical, Walnut Creek, CA, USA). Slides were incubated with antibodies targeting collagen-II, aggrecan, MMP-13, and ADAMTS-5, respectively. Histological images were analyzed under the BX53 microscope (Olympus). The grading score after histological staining was obtained by the criteria established by Masuda^43^. All image assessments were performed by 2 independent blinded observers.

#### **Statistical analysis**

Each experiment was repeated at least three times, and cells in every experiment were harvested from a single isolation process. Continuous data were expressed as mean ± standard deviation. Comparisons between two groups were performed by the Student’s *t*-test. Categorical data were analyzed by the chi-squared test. Prism version 7.0 (GraphPad Software, La Jolla, CA) or SPSS version 22.0 (SPSS Inc., Chicago, IL) was used for statistical analysis. Significance levels were set at *P < 0.05, **P < 0.01, and ***P < 0.001.

## Supplementary information

Supplementary Figure Legends

Supplementary Figure S1

Supplementary Figure S2

Supplementary Figure S3

Supplementary Table S1

Supplementary Table S2

Supplementary Table S3
