# Supplementary material for: The circular RNA FAM169A functions as a competitive endogenous RNA and regulates intervertebral disc degeneration by targeting miR-583 and BTRC
Source: Cell Death Dis. 2020 May 4;11(5):315. doi: 10.1038/s41419-020-2543-8 (PMC7198574; doi:10.1038/s41419-020-2543-8)
Supplement: Supplementary file 1 — Supplementary Figure Legends [file 41419_2020_2543_MOESM1_ESM.docx]

**Supplementary Figure Legends**

**Figure S1.** (a) The expression of circ-FAM169A was evaluated in NP samples from patients with or without IDD by RNA-FISH. The circ-FAM169A probe was labeled with Alexa Fluor® 488. Nuclei were stained with 4,6-diamidino-2-phenylindole (DAPI). Scale bar: 50 µm. (b) Quantitation of (a). Fluorescence was assessed in the IDD group relatively to the normal group. ***P<0.001.

**Figure S2.** Representative MRI images of various IDD grades

**Figure S3**. BTRC mRNA assessed by qRT-PCR in NP cells after transfection with circ-FAM169A or co-transfection with circ-FAM169A and miR-583. **P<0.001, ***P<0.001.
