# Supplementary figures and images for: The circular RNA FAM169A functions as a competitive endogenous RNA and regulates intervertebral disc degeneration by targeting miR-583 and BTRC
Source: Cell Death Dis. 2020 May 4;11(5):315. doi: 10.1038/s41419-020-2543-8 (PMC7198574; doi:10.1038/s41419-020-2543-8)

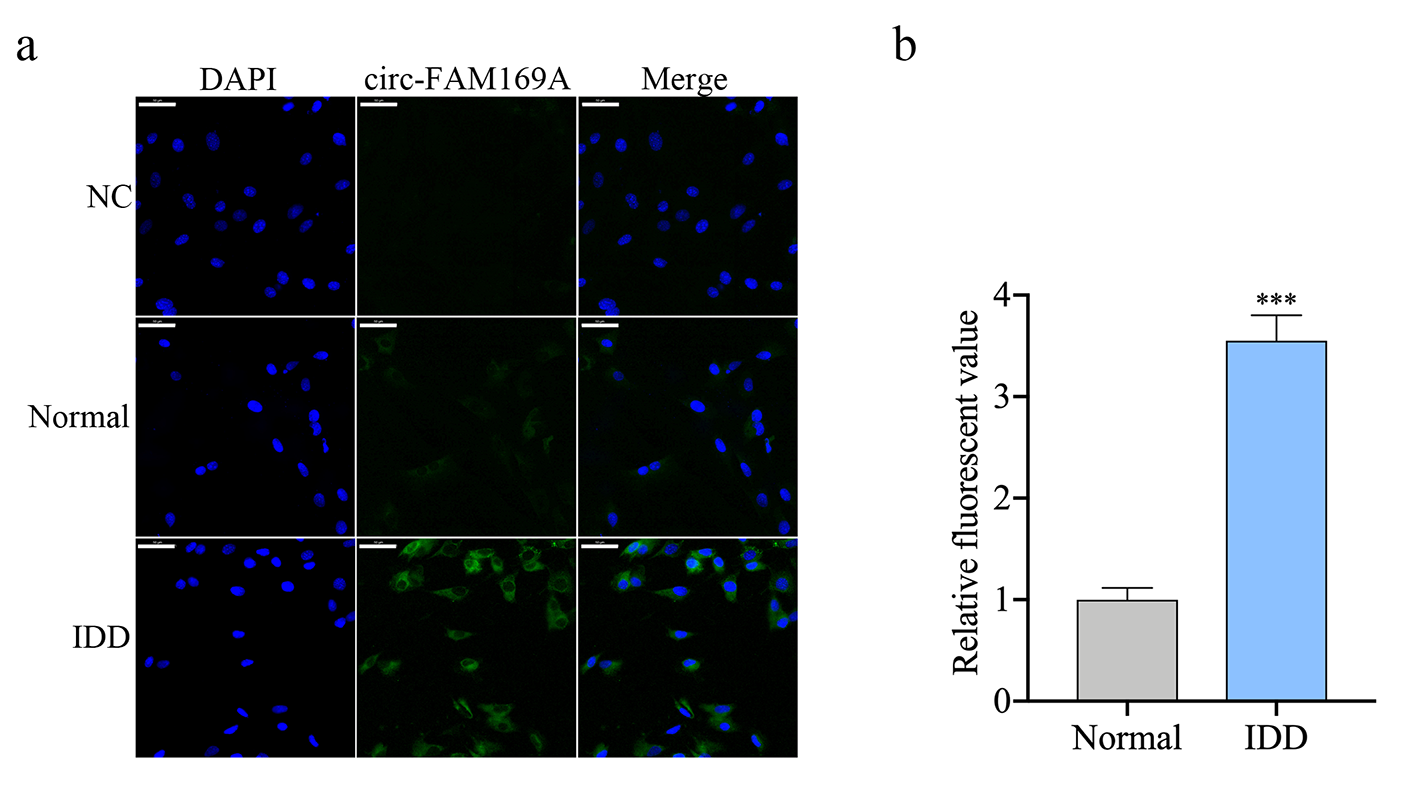

Supplement: Supplementary file 2 — Supplementary Figure S1 [file 41419_2020_2543_MOESM2_ESM.tif]

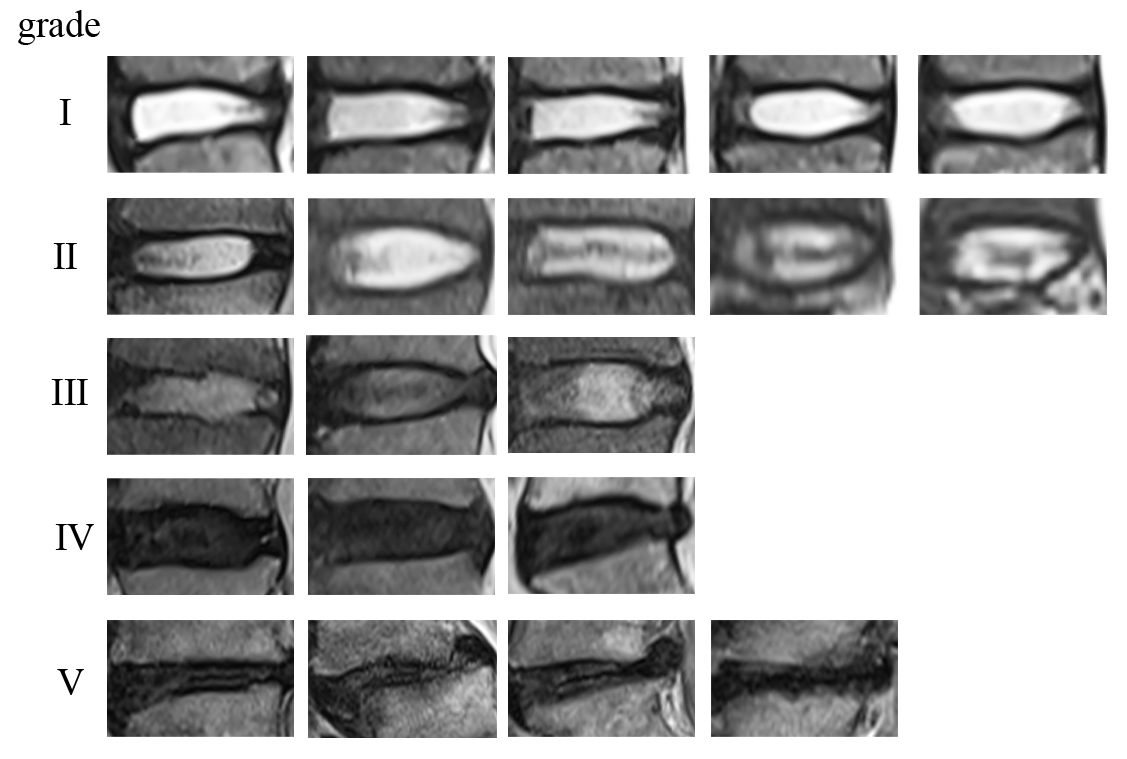

Supplement: Supplementary file 3 — Supplementary Figure S2 [file 41419_2020_2543_MOESM3_ESM.tif]

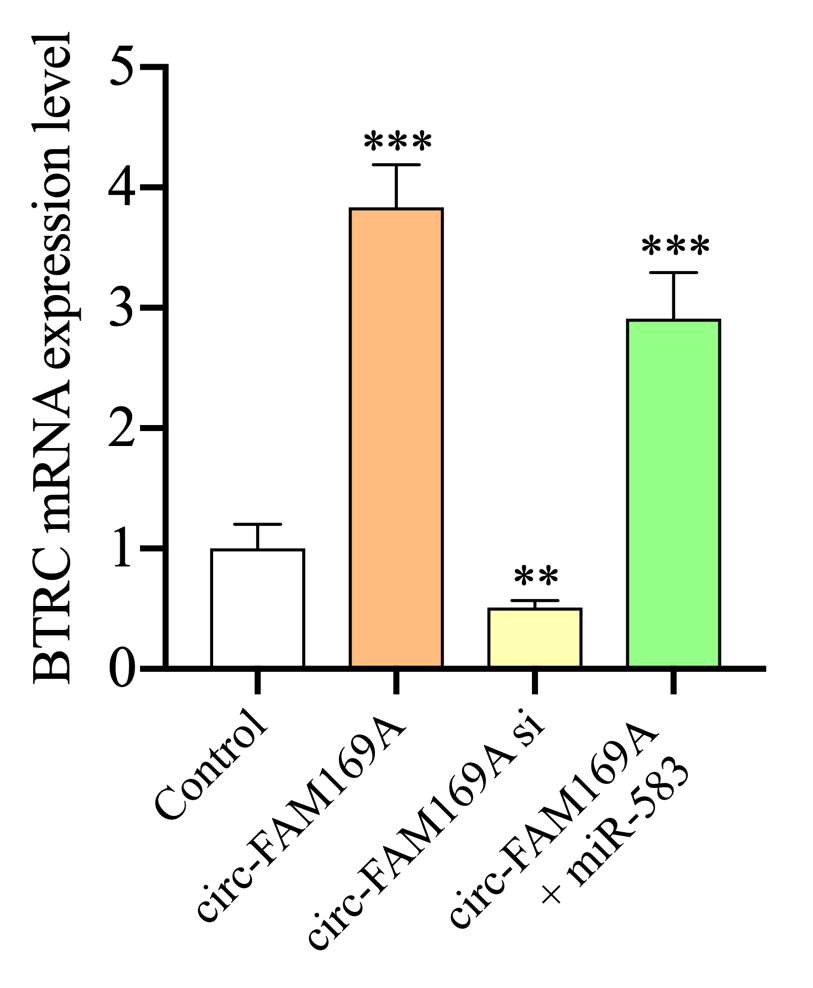

Supplement: Supplementary file 4 — Supplementary Figure S3 [file 41419_2020_2543_MOESM4_ESM.tif]
